# A Low Cost BLE Transceiver with RX Matching Network Reusing PA Load Inductor for WSNs Applications

**DOI:** 10.3390/s17040895

**Published:** 2017-04-19

**Authors:** Zhen Liang, Bin Li, Mo Huang, Yanqi Zheng, Hui Ye, Ken Xu, Fangming Deng

**Affiliations:** 1 School of Electronics and Information Engineering, South China University of Technology, Guangzhou 510641, China; zliang@rising-ic.com (Z.L.); phlibin@scut.edu.cn (B.L.); yqzhengee@scut.edu.cn (Y.Z.); hye@rising-ic.com (H.Y.); kxu@rising-ic.com (K.X.); 2 Rising Micro Electronics Co., Ltd., Guangzhou 510006, China; 3 School of Electrical and Automation Engineering, East China JiaoTong University, Nanchang 330013, China; dengfangming@ecjtu.jx.cn

**Keywords:** Bluetooth low power (BLE), wireless sensor networks (WSNs), TRX-switch, two-point modulation, single-ended LNA, ISM, transceiver

## Abstract

In this work, a low cost Bluetooth Low Energy (BLE) transceiver for wireless sensor network (WSN) applications, with a receiver (RX) matching network reusing power amplifier (PA) load inductor, is presented. In order to decrease the die area, only two inductors were used in this work. Besides the one used in the voltage control oscillator (VCO), the PA load inductor was reused as the RX impedance matching component in the front-end. Proper controls have been applied to achieve high transmitter (TX) input impedance when the transceiver is in the receiving mode, and vice versa. This allows the TRX-switch/matching network integration without significant performance degradation. The RX adopted a low-IF structure and integrated a single-ended low noise amplifier (LNA), a current bleeding mixer, a 4th complex filter and a delta-sigma continuous time (CT) analog-to-digital converter (ADC). The TX employed a two-point PLL-based architecture with a non-linear PA. The RX achieved a sensitivity of −93 dBm and consumes 9.7 mW, while the TX achieved a 2.97% error vector magnitude (EVM) with 9.4 mW at 0 dBm output power. This design was fabricated in a 0.11 μm complementary metal oxide semiconductor (CMOS) technology and the front-end circuit only occupies 0.24 mm^2^. The measurement results verify the effectiveness and applicability of the proposed BLE transceiver for WSN applications.

## 1. Introduction

There has been an explosive growth recently in wireless sensor networks (WSN) [1], whose applications have been extended to autonomous health monitoring, remote or hazardous area monitoring, and emergency management. The sensor nodes in these networks are typically connected to multiple types of devices, including smartphones, wearables and PCs [2]. For the sake of flexibility, low cost and seamlessness, the communication among these nodes requires the use of a commonly or easily available wireless technique. Therefore, Bluetooth Low Energy (BLE) is a competitive candidate [3,4] because of its already massive establishment in the mobile market. 

Due to the limited energy source (e.g., battery or harvested energy) of the sensor nodes, an ultra-low power (ULP) transceiver design for WSN applications is highly desirable. In addition, because of the stringent market requirements, there has been a strong drive in recent years to decrease the cost and module area of wireless sensor nodes [5]. This can be achieved through shrinking the silicon area and reducing the external components. Therefore, the investigation on low power and low cost BLE transceivers is indispensable. 

**Figure 1:**
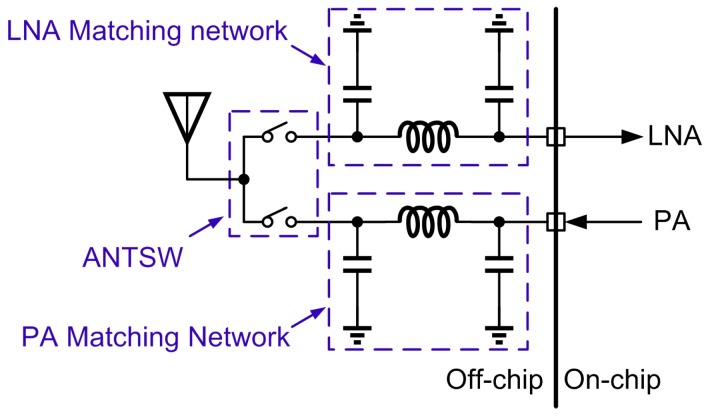
The conventional RF front-end configuration.

For a conventional radio frequency (RF) front-end configuration [6] as shown in Figure 1, there are several external components: (1) the impedance matching network for the low noise amplifier (LNA); (2) the TRX-antenna switch; and (3) impedance matching network for the power amplifier (PA). This leads to an increase in the printed circuit board (PCB) area and cost. For a low cost BLE module, these external TRX-switches and matching networks [6,7] are clearly not favorable. To achieve higher integration, an on-chip CMOS TRX-switch technique has been reported in [8], yet the non-linearity and insertion loss introduced by CMOS transistors will deteriorate the performance of the transceiver. In [9,10,11], an on-chip balun shared by the transmitter (TX) and receiver (RX) was used to convert the single-ended signal to a differential one, and eliminated the need of a separated TRX-switch through the principle of impedance conversion. However, the differential architectures for both LNA and PA is power-consuming for an ultra-low power transceiver. Meanwhile, the insertion loss of the on-chip balun will degrade RX noise figure (NF). In [12,13], a TRX-switch and matching network were integrated using multiple inductors, which is area-consuming and thus not favorable in low cost design.

To address the aforementioned issues, a low power and low cost transceiver for 2.4 GHz ISM band was presented in this work. Seeking a low-power implementation, a single-ended LNA and PA were employed in the transceiver. In order to further reduce the chip cost and module area, a front-end with a RX matching network reusing PA load inductor was proposed. 

This work is organized as follows: the front-end circuit implementation of the transceiver is described in Section 2. Section 3 shows other circuit implementations. The measurement results and discussion are described in Section 4, and conclusion is drawn in Section 5.

## 2. Proposed Front-End with RX Matching Network Reusing PA Load Inductor

An integrated TRX-switch/matching network presents some design challenges. Firstly, reliability issues will be introduced by the large PA output power through stressing the low noise amplifier (LNA) input transistors. This requires an input voltage swing reduction of LNA at TX mode, and thus a large RX off-state impedance (Z_RX-off_). Secondly, the TX performance features, such as output power, error vector magnitude (EVM) should not be degraded by the Z_RX-off_. Similarly and finally, the off-state TX impedance (Z_TX-off_) should not deteriorate RX performance such as NF and linearity. 

BLE specification asks for only −70 dBm sensitivity (1-MHz channel bandwidth) at GFSK modulation with a required SNR of 14 dB [4]. The NF can thus be obtained by:

NF = −(−174 dBm/Hz) − 10 × Log(BW) − SNR_out_ + Sensitivity, (1)

where BW and SNR_out_ represent channel bandwidth and required demodulator signal-to-noise ratio (SNR), respectively. The maximum allowable NF obtained using (1) is 30 dB. However, in order to increase the link budget, a NF below 7 dB was targeted in this work. This allows a reasonable matching network design trade-off between RX noise and power delivery.

**Figure 2:**
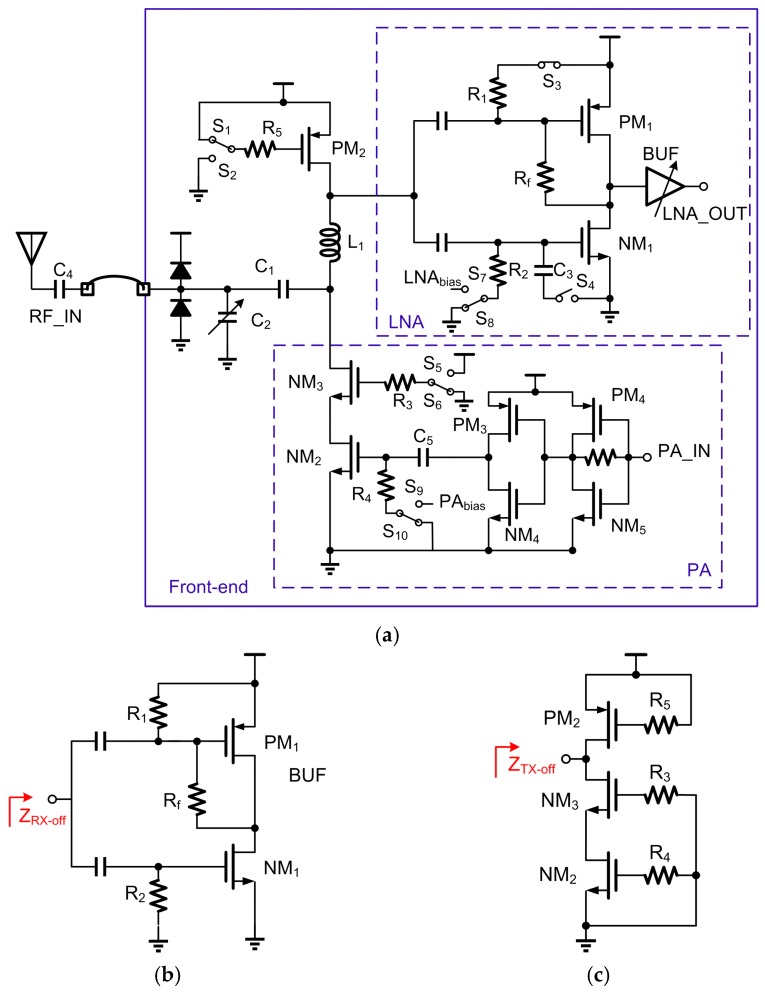
(**a**) Proposed front-end with RX matching network reusing PA load inductor; (**b**) Simplified LNA circuit in TX mode; (**c**) Simplified PA circuit in RX mode.

Figure 2a shows the simplified schematic of the proposed front-end with RX matching network reusing PA load inductor. The front-end integrated the TRX-switch and matching network. The PA load inductor L_1_ is reused as the impedance matching component for RX. Capacitors C_1_ and C_2_ are employed to match the impedance in both RX and TX paths. Switches S_1_–S_9_ are adopted to determine the mode of the front-end circuit. 

In TX mode, S_1_, S_4_, S_7_, S_5_ and S_9_ are turned off while S_2_, S_3_, S_5_, S_8_ and S_9_ are turned on. The inductor L_1_ acts as the load of the PA which is typically supplied by DC-DC convertor through the PM_2_. For PM_2_ size, it is determined by the following three factors: (1) The on-resistance. Large on-resistance causes a large voltage drop, and thus reduces the efficiency of PA. (2) The parasitic resistance and capacitance when PM_2_ is turned off. RX NF might thus be degraded. (3) The current density. The size of PM_2_ should be large to accommodate large PA current. Therefore, the size of PM_2_ is chosen to be 0.11 μm/200 μm, where the width is 5 μm and the finger number is 40. For LNA, the input transistors NM_1_ and PM_1_ are turned off and the simplified circuit is shown in Figure 2b. Consequently, the PA sees the impedance of the LNA path (Z_RX-off_) as a small capacitor shunted with a large resistor given as R_RL_‖1/jωC_RL_. Around 2.45 GHz, simulation shows the real and imaginary parts of Z_RX-off_ are 2.6 kΩ and 95 fF, respectively. Simulation shows about 4% PA efficiency loss due to Z_RX-off_ and PM_2_.

In RX mode, S_1_, S_4_, S_7_, S_5_ and S_9_ are turned on and S_2_, S_3_, S_5_, S_8_ and S_9_ are turned off. For PA, the transistors (NM_2_, NM_3_ and PM_2_) are turned off and the simplified circuit is shown in Figure 2c. Similarly, LNA sees PA path as a large impedance (Z_TX-off_), consisting of a small capacitor shunted with a large resistor and can be given as Z_TX-off_ = R_TL_‖1/jωC_TL_. Around 2.45 GHz, the simulated real and imaginary parts of Z_TX-off_ are 16 kΩ and 66 fF, respectively. This large impedance Z_TX-off_ results in a 0.4 dB insertion loss, which is acceptable for the NF requirement of BLE.

### 2.1. PA and PA Matching Network

In the TX, a switching-type single-ended PA operating in non-linear region is employed for the constant envelope modulation required by BLE, as shown in Figure 2a. The PA is typically powered by a DC-DC convertor with reduced output noise [14,15] for better PA linearity. This PA composes of three stages of amplification, with two inverters as pre-amplifiers for the gate voltage of NM_2_. For the first one, it is placed in proximity to VCO to drive the long signal path between VCO and PA. For the second one, it does not generate a rail-to-rail output signal. The final stage is a cascode-type amplifier, where NM_2_ adopts thin gate oxide to obtain input/output isolation. The cascode stage NM_3_ increases the output impedance of the PA and employs thick gate oxide to withstand peak drain voltage beyond power supply due to the inductive load [16]. The size of the second inverter, together with NM_2_ size and NM_2_ gate bias, are carefully chosen to obtain the input signal of NM_2_ with appropriate amplitude and operation point, which reduce 2nd harmonic component while maintain the required output power. Simultaneously, 2nd harmonic component is further attenuated by 15 dB with the matching network.

**Figure 3:**
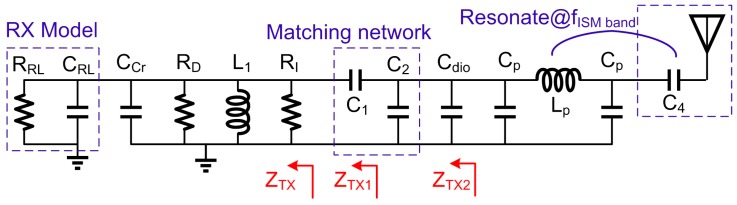
TX matching network analysis.

**Figure 4:**
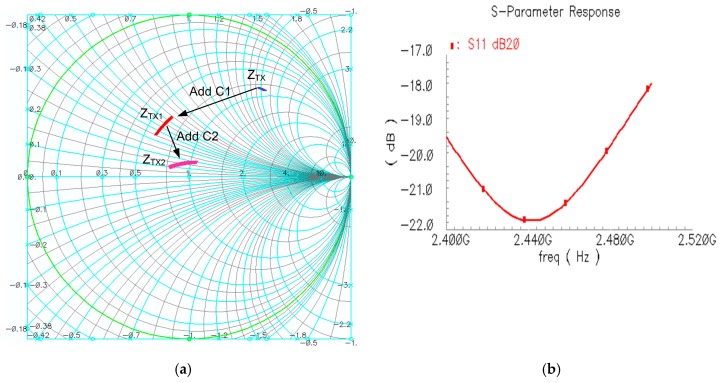
TX impedance analysis. (**a**) TX impedance movement in Smith chart; (**b**) The final S_11_ of TX impedance from 2.4 GHz to 2.5 GHz.

In TX mode, the simplified small signal equivalent circuit diagram of Z_TX_ is shown in Figure 3, and the output impedance can be written as:

(2) $$Z_{TX}=j\omega L_{1}||R_{D}||R_{I}||\frac{1}{j\omega C_{cr}}||Z_{RX-off},$$

(3) $$R_{D}=\left[1+(g_{m2}+g_{mb2})r_{o3}\right]r_{o2}+r_{o3},$$

where R_D_ represents the output impedance of the cascode NM_2_ and NM_3_, R_I_ represents the shunt resistance of the inductor, C_cr_ represents the sum of various parasitic capacitances seen from the output. The value of L should be large to increase output power and make Z_TX_ in inductive region in Smith chart, as shown in Figure 4a. Therefore, Z_TX_ can be simplified as a resistor R_t_ shunted with an inductor L_t_, and the admittance Y_TX_ can be written as:

(4) $$Y_{TX}=\frac{1}{R_{t}}-j\frac{1}{\omega L_{t}}=(\frac{1}{R_{D}}+\frac{1}{R_{I}}+\frac{1}{R_{R}{}_{L}})-j\left[\frac{1}{\omega L_{1}}-\omega C_{c}{}_{r}-\omega C_{RL}\right],$$

Two additional matching components help to match Z_TX_ to 50 Ω. Taking the compatibility with the RX matching network (mentioned below) into account, the matching network of a series capacitor C_1_ and a shunt capacitor C_2_ is selected. Z_TX1_ is the series impedance of Z_TX_ and C_1_, and Y_TX2_ is the shunt admittance of Z_TX1_ and C_2_, which are written as:

(5) $$Z_{TX1}=\frac{jR_{1}\omega L_{t}}{R_{t}+j\omega L_{t}}+\frac{1}{j\omega C_{1}},$$

(6) $$Y_{TX2}=\frac{1}{Z_{TX1}}+j\omega C_{2}+j\omega C_{dio},$$

where C_dio_ represents the capacitance introduced by ESD diode. From (5) and (6), the value of C_1_ and C_2_ can be obtained if the output impedance is matched to 50 Ω, and Figure 4a shows the trajectory of the Z_TX_ when applying C_1_ and C_2_. Another factor needs to be considered is bonding wire which can be simply modeled as two capacitors shunted with an inductor. The inductance of the bonding wire can be cancelled by its resonating with the output decoupling cap C_4_ at 2.45 GHz. As shown in Figure 4b, the overall simulated TX impedance S_11_ is smaller than −18 dB within in ISM band. 

### 2.2. LNA and LNA Matching Network

To allow the RX a good coexistence performance with an in-band blocker [9], the LNA and mixer should be designed to be linear to prevent these blockers from degrading the performance. As shown in Figure 2, the LNA adopts a push-pull common-source inductorless topology [17], which inherently achieves higher linearity and power-efficiency when compared with a conventional NMOS-only LNA [18]. 

**Figure 5:**
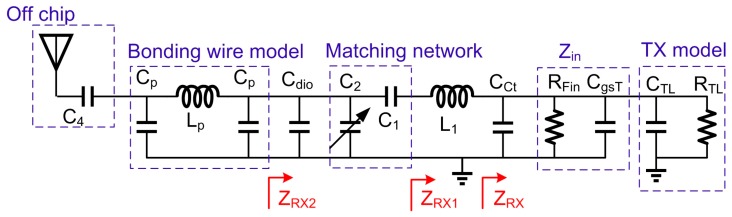
RX matching network analysis.

In RX mode, the simplified small signal equivalent circuit diagram of *Z_RX_* is shown in Figure 5, and the output impedance can be written as:

(7) $$Z_{RX}=Z_{in}||\frac{1}{j\omega C_{c}{}_{t}}||Z_{TX-off},$$

where C_ct_ represents the sum of all of parasitic capacitances seen from the LNA input, *Z_in_* represents the LNA input impedance and can be expressed as [17]:

(8) $$Z_{in}=\frac{R_{Fin}}{1+(R_{Fin}C_{\omega }^{gsT}{}^{2})}-j\frac{R_{Fin}{}^{2}C_{gsT}\omega }{1+{\left(R_{Fin}C_{gsT}\omega \right)}^{2}},$$

(9) $$R_{Fin}=\frac{R_{f}}{1+\left|G_{V}\right|},$$

(10) $$C_{gsT}=\;C_{3}+\;C_{gsn1}+C_{gsp1},$$

where *C_gsn_*_1_ and *C_gsp_*_1_ represent the capacitance between the gate and the source of NM_1_ and PM_1_, respectively. G_V_ represents the LNA voltage gain and can be written as:

(11) $$G_{V}=\frac{1}{2}\frac{g_{mT}}{R_{s}C_{gsT}\omega_{0}}(r_{ds1}||r_{ds2}||R_{f}||R_{Mixin}),$$

(12) $$g_{mT}\;=\;g_{mn1}\;+\;g_{mp1}$$

Here *g_mn_*_1_ and *g_mp_*_1_ represent the transconductance of NM_1_ and NM_2_ respectively, and *R_s_* is 50 Ω. Thus, *Z_RX_* can be simplified as a resistor *R_r_* shunted with an inductor *C_r_*, and the admittance *Y_RX_* can be expressed as:

(13) $$Y_{RX}=\frac{1}{R_{r}}+j\omega C_{r}=(\frac{1}{R_{Fin}}+\frac{1}{R_{RL}})+j\left[\omega (C_{gsT}+C_{c}{}_{t}+C_{T}{}_{L})\right]$$

Considering there is no inductive device in the LNA circuit, Z_RX_ is in the capacitive region in Smith chart. Therefore, the PA load inductor L_1_ is reused in RX matching network. As described above, *L*_1_ moves *Z_RX_* to the inductive region in Smith chart. *Z_RX_*_1_ represents the series impedance of *Z_RX_* and *L*_1_, which can be written as:

(14) $$Z_{RX1}=\frac{R_{r}}{1+{\left(\omega R_{r}C_{r}\right)}^{2}}-j\frac{\omega R_{r}C_{r}}{1+{\left(\omega R_{r}C_{r}\right)}^{2}}+j\omega L_{1}.$$

**Figure 6:**
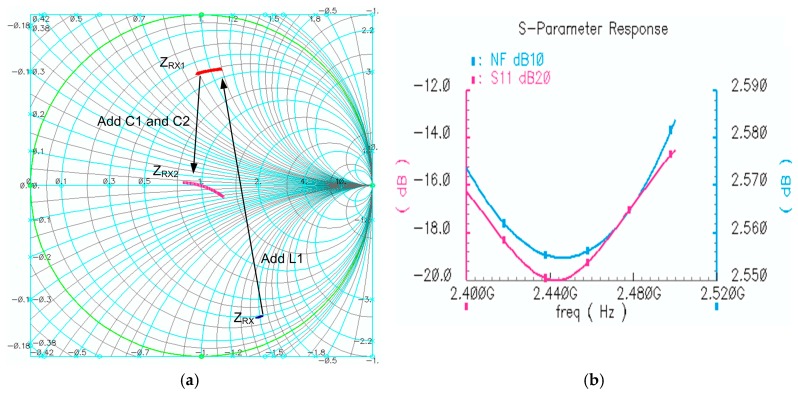
(**a**) RX impedance movement in smith chart; (**b**) The final NF and S_11_ of RX impedance from 2.4 GHz to 2.5 GHz.

Then *Z_RX_*_1_ can be moved to near the center point via the capacitor *C*_1_ and trimming the capacitor *C*_2_. Figure 6 shows the trajectory of the *Z_RX_* when applying *L*_1_, *C*_1_ and *C*_2_. *Z_RX_*_2_ represents the impedance after adding *C*_1_ and *C*_2_, and can be expressed as:

(15) $$Z_{RX2}=(Z_{RX1}+\frac{1}{j\omega C_{1}})||\frac{1}{j\omega （C_{2}+C_{dio})}.$$

As shown in Figure 6b, the simulated LNA NF is around 2.57 dB and the maximum S_11_ is about −15 dB.

### 2.3. The Summarized Merits of this Proposed Frond-End

There are some merits of the proposed front-end with RX matching network reusing PA load inductor: (1) Only one inductor is employed in this front end. It is used for PA load in TX mode, and reused as a matching component in RX mode. Thus, the chip area has been reduced significantly; (2) The front-end has integrated the TRX-switch and matching network, and reduced the number of external components and thus reduced the cost of WSNs nodes; (3) RX and TX performance have not been deteriorated: for one, the impedance of LNA and PA can be optimized respectively using the proposed scheme; for the other thing, nonlinear components, such as CMOS switch, have not been adopted in the signal path of matching network; (4) It is free of TX reliability issues, because the PA sees the RX path as a high impedance compared with 50 Ω and the input voltage swing of the LNA is very small.

## 3. Transceiver Circuit Implementation

### 3.1. Transceiver Architecture

Recent designs for the 2.4 GHz ISM band receiver employed either the sliding-IF [19,20,21], direct conversion [22,23] or low-IF architecture [9], which have been demonstrated to be the most feasible to meet BLE performance requirements under severe power and chip area constraints. The sliding-IF architecture shifts the RF signal into the analog baseband (ABB) signal with twice frequency conversions [24,25]. This architecture facilitates LO generation and distribution at a favorably lower frequency, but causes a systematic, difficult-to-avoid susceptibility to out-of-band (around 1.45 GHz) image interference. Furthermore, GFSK modulation used in BLE contains significant energy at very low frequencies close to DC. Considering direct conversion architecture is susceptible to DC offset and flicker noise, low-IF architecture is adopted in this work. 

**Figure 7:**
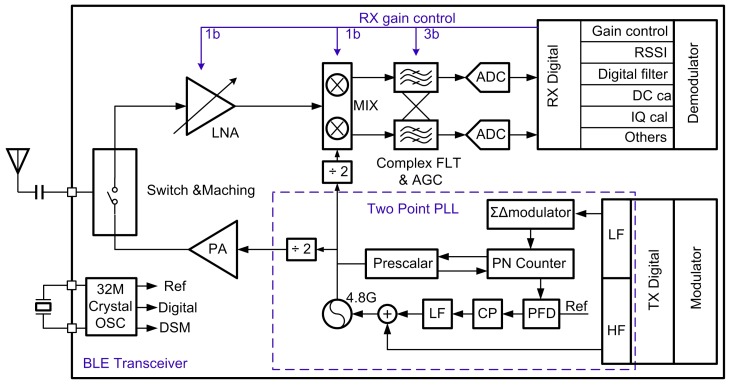
The proposed BLE TRX architecture.

Figure 7 shows the simplified block diagram of the proposed BLE transceiver. It includes the aforementioned TRX-switch and matching network integrated front-end, a low IF RX, a two-point modulation based TX, a fractional-N synthesizer, digital modulator and demodulator, and several digital signal processing. 

The RF signal passes first through the integrated TRX-switch and matching network, in which the inductor is shared between RX and TX. The on-chip switch drives the single-end variable-gain LNA, which amplifies the RX signal before it is fed to a current bleeding quadrature down conversion mixer. A 4th order complex band pass filter (BPF) follows the mixer, which performs channel selection, image rejection and anti-aliasing filtering, and also serves as a programmable gain amplifier. The IF signal is then digitized by a 3rd order delta-sigma continuous time (CT) ADC [26]. After that, the signals are further processed in the digital sections, such as additional channel select digital filtering, RSSI estimation, dc offset cancellation, automatic gain control (AGC), IQ imbalance calibration, and demodulation [27].

For BLE intermodulation characteristics, the required signal shall be measured at a power level of 6 dB over the reference sensitivity level [4]. The minimum BLE requirements for the input 3rd intermodulation point (IIP_3_) and input 2nd intermodulation point (IIP_2_) can be calculated as follows [28]. IIP_3_ (min) = Pin + 1/2 × (P_1_ − P_3_). Where P_in_ = −50 dBm, P_3_ = −70 (sensitivity) + 6 (dB) −14 (SNR) = −78 dBm and P_1_ = −50 dBm. Thus, IIP_3_ (min) = −36 dBm. A −22 dBm IIP_2_ (min) is calculated in a similar way. However, in order to increase the link margin, IIP_3_ above −20 dBm and IIP_2_ above 0 dBm was targeted in this work.

BLE TXs usually adopt two architectures: conventionally mixer-based [29], and two-point PLL-based TX [22]. The mixer-based TXs can support universal modulations, but at the expense of high circuit complexity and power consumption. Additionally, the severe PA-to-VCO coupling in mixer-based TXs [30] gives rise to frequency pulling effect, which degrades the TX modulation accuracy and increases output spectral regrowth [31,32]. In this work, the TX employed a two-point PLL-based architecture with a non-linear PA to eliminate the power-hungry RF mixers and quadrature local oscillator (LO) generators, as shown in Figure 7. High frequency (HF) and low frequency (LF) data paths are produced from digital domain to directly modulate a fractional-N synthesizer working at two times carrier frequency. LF acted as the first point modulation which can be modulated by a slowly-varying frequency modulation (FM) signal. On the other hand, HF extended the FM bandwidth to beyond the PLL bandwidth, which is the second point modulation. After dividing by 2, the modulated signal is directly fed to a nonlinear PA. Although the specified minimum output power of BLE standard is −20 dBm [4], the 0 dBm transmit power is targeted in this work. 

The PLL phase noise not only affects the EVM of RX and TX, but also affects the interference and intermodulation performance. For the BLE specification [4], the interference performance shall be measured with a wanted signal 3 dB over the reference sensitivity level. The minimum BLE requirements for phase noise at offset 3 MHz can be calculated as:

PN_(max)_@3M = −70 dBm + 3 − 14 (SNR) − P(blocker) − 10 × log(1M) = −106 dBc/Hz (16)

where P(blocker) is the adjacent (3 MHz) interference which is 27 dB higher than the wanted signal, and 10 × log(1M) is the logarithm of 1 M transmitted bandwidth. However, in order to increase the link budget, the phase noise at offset 3 MHz was designed to below −110 dBc/Hz in this work.

### 3.2. Down Conversion

**Figure 8:**
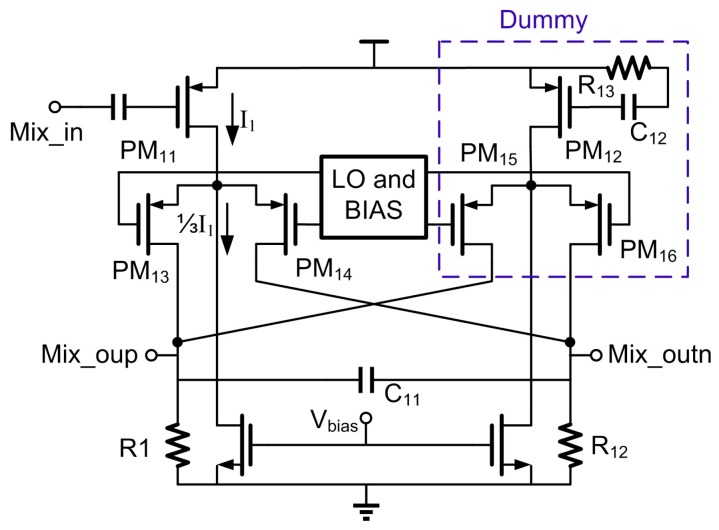
Simplified schematic of down conversion.

Figure 8 illustrates one path of the quadrature mixer. Although the popular passive mixer [9] can save current in mixer stage, it needs large current to drive LO to full swing, and thus it is not considered in this work. Acting as a Gilbert type, this mixer employed PMOS as input and switch transistors for smaller flick noise. The dummy mixer renders the mixer symmetric from the LO standpoint and enhances IIP_2_. The square root currents of the input PFETs PM_11_ and PM_12_ are proportional to the mixer linearity, while the larger current, the larger LO swing is required for the switching transistors. Meanwhile, increasing the switching current will increase the switching stage noise. In order to solve the trade-offs among noise, linearity and power consumption, the current-bleeding prototype was employed in this work. To minimize the differences in the two halves caused by the single-end output of LNA, the gate of PM_12_ in dummy mixer is connected to a resistor R_13_ and capacitor C_12_, which matches the output impedance of the single LNA at the desired RF frequency. The resistors R_11_ and R_12_ not only act as the mixer load, but also serve as roofing filter together with capacitance C_11_. This filter implements the first channel filtering pole in the RX path. 

### 3.3. LPF and ADC

**Figure 9:**
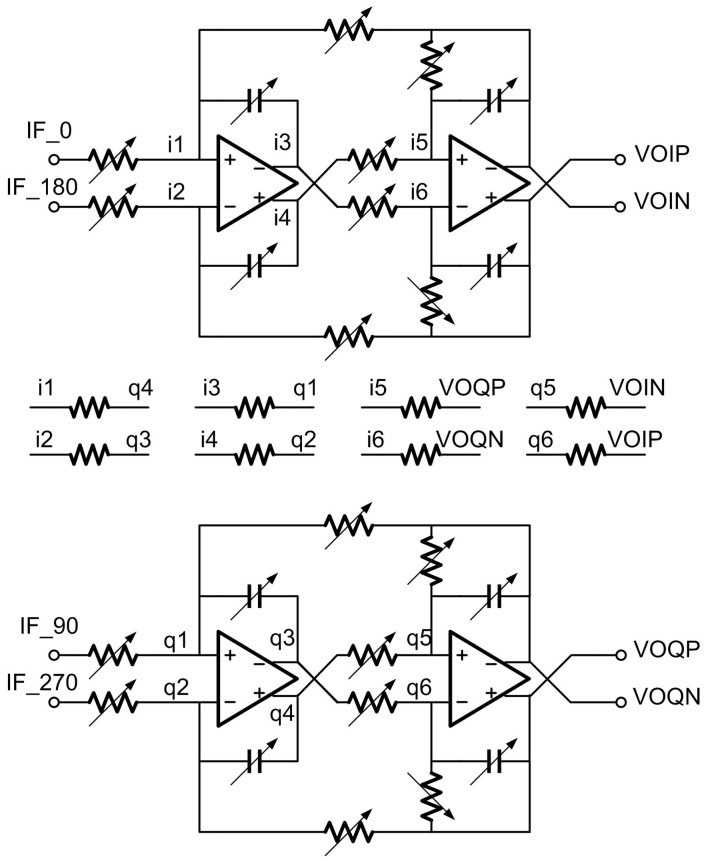
The simplified schematic of a 2nd order complex filter.

Following the mixer, out-of-band blockers and image interferences are filtered out sufficiently by the analog baseband 4th Butterworth active-RC complex filters. This 4th order complex filter is achieved with two 2nd order filters cascaded, and the simplified schematic of the 2nd order complex filter is given in Figure 9. 

Compared with the real BPF, complex filter provides rejection at the image channel. In addition, the complex filters act as programmable gain amplifiers, with gain range from 2 to 20 dB controlled by the digital module. This relatively low voltage gain prevents the DC-offset saturating the coming stages even without the DC-offset cancellation circuits. Though an active-RC circuit is commonly used as the complex filter, its frequency characteristics vary with the RC time constants, which are likely to change due to power, voltage and temperature (PVT) variations. To handle this issue, RC constant time calibration circuits [33] is added in this work.

**Figure 10:**
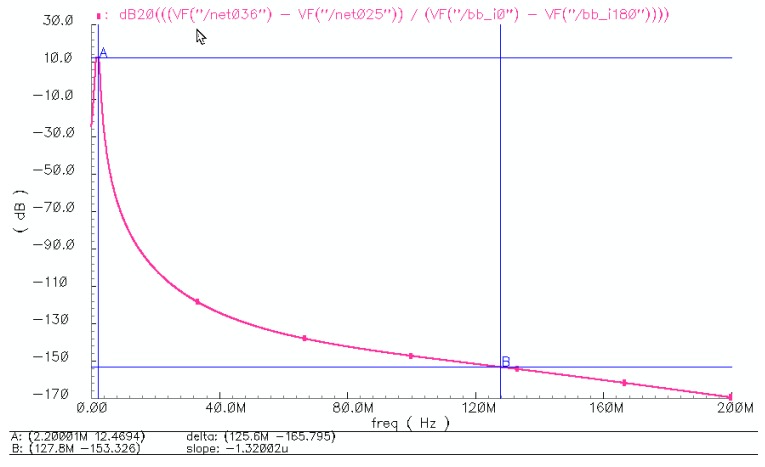
The simulated magnitude-frequency response of the 4th order complex filter.

The complex filters also provide anti-alias filtering for the 3rd order delta-sigma CT ADC, which is sampled with a 128 MHz clock. The simulated magnitude-frequency response of the 4th order complex filter is shown in Figure 10, that more than 150 dB attenuation at 128 MHz is obtained, which avoid aliasing issue. The CT-ADC loop coefficients employ active-RC networks and can be digitally tuned using the same code generated by the RC constant time calibration circuits in complex filter.

### 3.4. Synthesizer

As shown in Figure 7, the LO is generated by a 4.8 GHz fractional-N synthesizer [34], consisting of a phase-frequency detector (PFD), a charge pump (CP) current source, a loop filter, a third order multi-stage noise shaping (MASH) delta-sigma modulator (DSM), a dual-modulus prescaler, programmable dividers, and a voltage-controlled oscillator (VCO). Considering the phase noise and respond time, the PLL loop bandwidth of 120 kHz was adopted in this work. However, this loop bandwidth is significantly smaller than the BLE specified 1 Mb/s rate with GFSK modulation. To extend the modulation bandwidth, a two-point modulation scheme is applied to reuse the synthesizer. This simultaneously guarantees reduced noise performance in both TX and RX modes.

**Figure 11:**
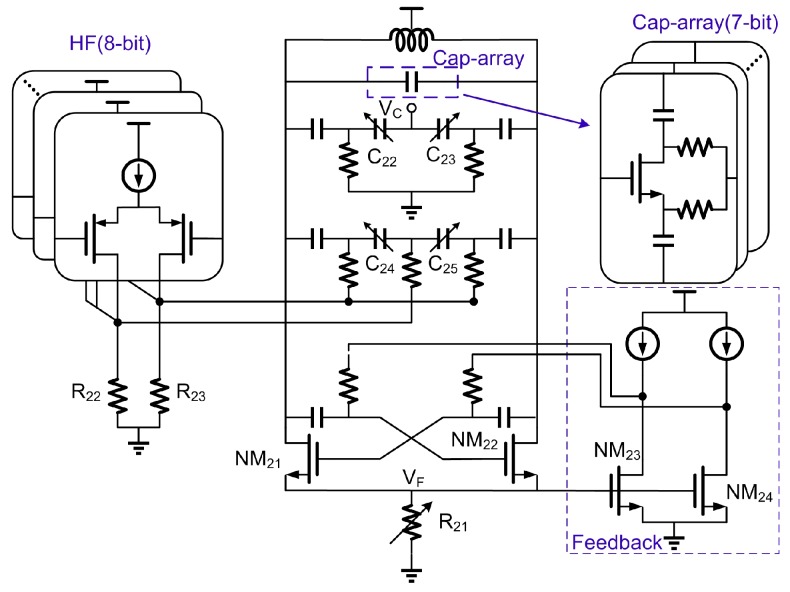
Simplified schematic of VCO.

Figure 11 shows the simplified VCO schematic. The NMOS only cross-coupled topology is employed due to its better phase noise performance [35] in low power design. The charge pump output is applied to MOS-varactors C_22_ and C_23_ for fine frequency and phase-locking after the 3-order loop filter. To improve the linearity of the VCO gain (Kvco), both C_22_ and C_23_ were designed to have two combined varactors biased to ground. An 8 bit DAC is added as HF (the second point) to extend the frequency modulation bandwidth beyond the PLL bandwidth. The designed frequency resolution is about 2 kHz. A symmetric and differential customized inductor is adopted to increase the Q-value and reduce current consumption. Current biasing is achieved by a variable poly resistor R_21_, which suffers lower noise as comparing to a tail current source. The output impedance of NM_21_ and NM_22_ are increased through NM_23_ and NM_24_ by using negative feedback, which makes the node (V_F_) voltage be less sensitive to external interference and provide steadier current for resonance device. Therefore, the phase noise of the VCO is much less sensitive to bias noise in low frequencies [23].

To cover frequency range from 4.8 GHz to 4.967 GHz (two times of ISM band frequency), a 7-bit capacitor array was used as shown in Figure 11. In order to speed up the lock time, initial coarse frequency algorithm is implemented in digital domain during startup period. The control word of the capacitor array is scanned and the corresponding frequency is calculated and stored. When channel switching, the required control word will be automatically picked up to achieve a fast lock. After the PLL has settled to the channel frequency, the HF data is then applied to other MOS varactors consisting of C_24_ and C_25_ in TX mode.

## 4. Measurement Results and Discussion

**Figure 12:**
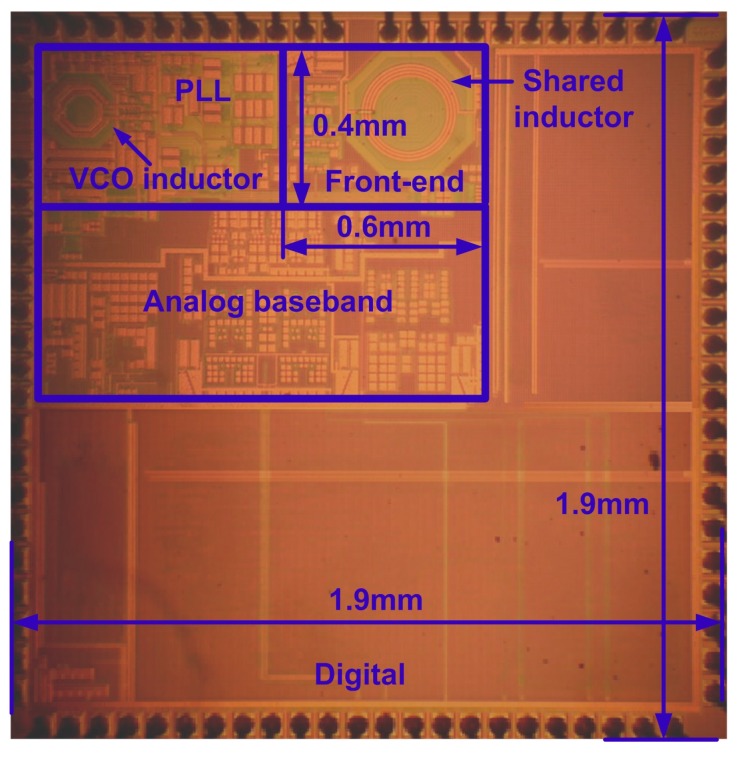
Chip micrograph of the proposed BLE transceiver.

The BLE transceiver is implemented in a standard 110 nm CMOS technology using a single-poly and six-metal layers (one thick copper and one thick aluminum layer). The chip microphotograph is shown in Figure 12. Only two inductors, one for front-end and one for VCO, were implemented. The die area is 3.6 mm^2^, in which the front-end circuits only occupies 0.24 mm^2^ , while the rest is PLL, analog baseband, digital modular, demodulator and digital signal processing. Without using off-chip TRX-switch and Balun, this transceiver achieves small area and high-level integration. 

**Figure 13:**
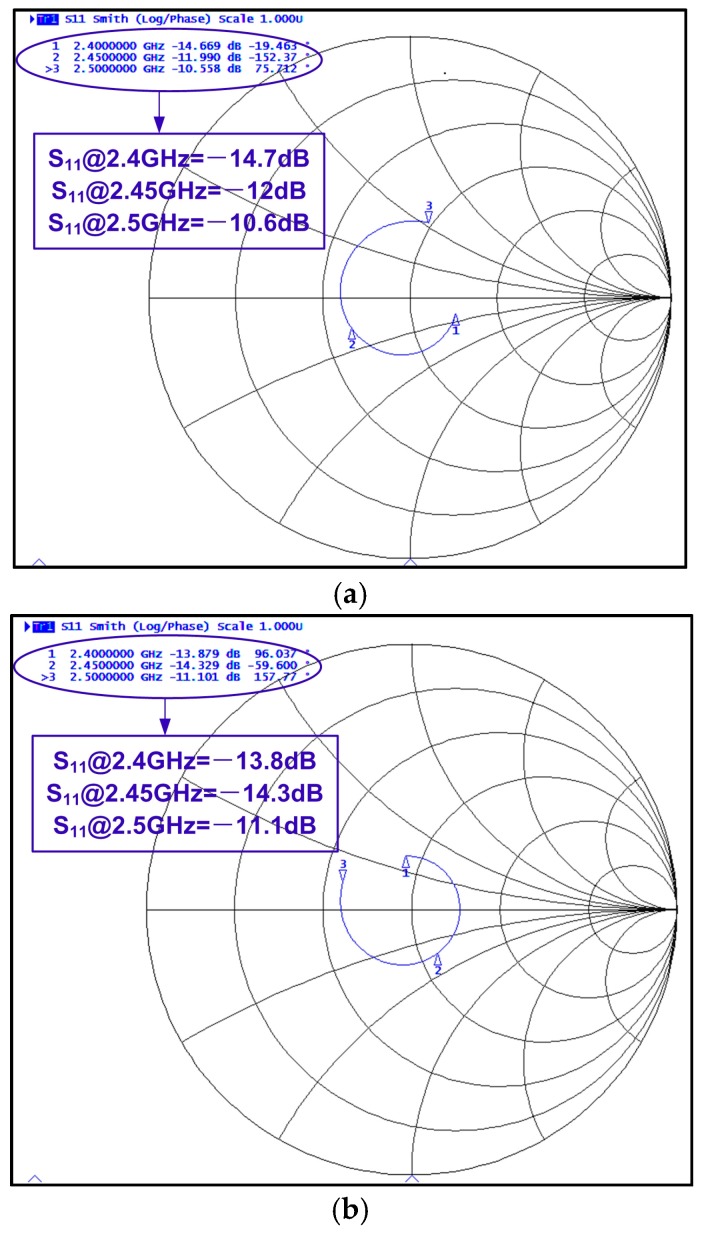
(**a**) Measured TX impedance in SMITH chart; (**b**) Measured RX impedance in SMITH chart from 2.4 GHz to 2.5 GHz.

Figure 13 shows the S_11_ of the RX and TX. Both TX and RX achieve S_11_ less than 10 dB over the entire operating band. This verifies the effectiveness of the proposed TRX-switch and matching network combination scheme. The discrepancy between the measured S_11_ and simulated S_11_ (shown in Figure 4 and Figure 6) might result from the inaccurate modeling from both inductor and the parasitic components of PCB and bonding wire. 

**Figure 14:**
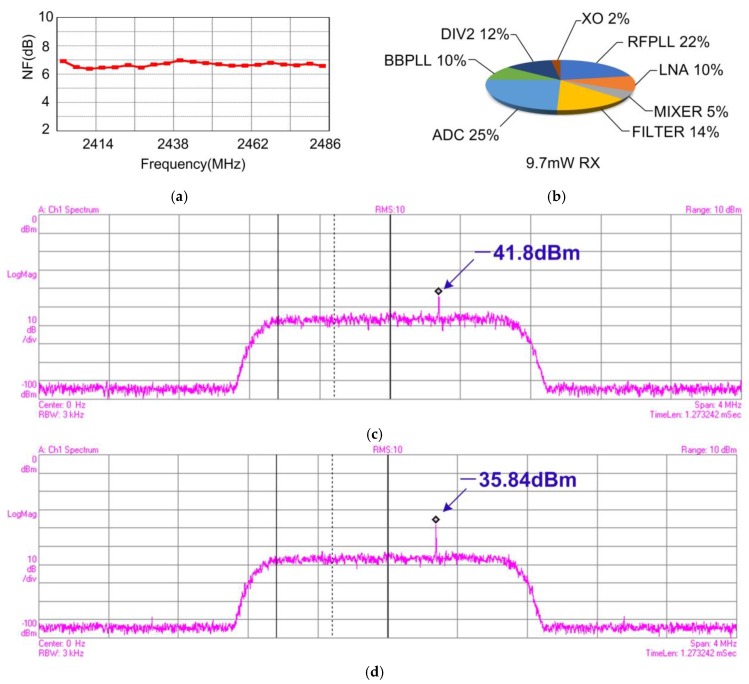
(**a**) Measured NF in ISM band; (**b**) RX power loss breakdown; (**c**) Measured the third-order intermodulation product; (**d**) Measured the two-order intermodulation product.

The noise performance of the RX has been evaluated from the antenna port to the ADC outputs. Figure 14 shows the measured RX performance. The NF of the RX shown in Figure 14a achieves 6–7 dB within the ISM band. Therefore, the sensitivity for the BLE Standard calculated from (1) is about −93 dBm, which is much higher than the BLE specification requirements (−70 dBm). The total current consumption of RX is 9.7 mW, and the power loss breakdown of RX is shown in Figure 14b. Among the RX power losses, the ADC and complex filter consume 2.4 and 1.4 mW, respectively, which are mainly from their operational amplifiers. LNA consumes 1 mW, 70% of which is from the first stage, while the buffer contributes the remaining 30%. The mixer consumes less than 0.5 mW which is mainly from the core circuits. 1 mW is consumed by BBPLL, 40% of which is from the ring oscillator, 20% from PFD, 20% from CP, the rest from divider. RFPLL consume about 2.1 mW of power, with its VCO, PFD, CP and prescalar consuming 1 mW, 0.2 mW, 0.2 mW and 0.6 mW, respectively. 

For the linearity performance, the measured 1 dB compression point (P_1dB_) of the receiver is about −29 dBm. Additionally, Figure 14c,d show the 3rd and 2nd intermodulation products. Two interfering sine wave signals (listed in Table 1) are input to the receiver with a channel gain G_C_ = 74 dB, and IIP_3_ = P_in_ + (P_in_ + G_C_ − P_O3_)/2, while IIP_2_ = 2P_in_ − P_O2_ + G_C_. The measured IIP_3_ and IIP_2_ are thus −17.1 dBm and 9.8 dBm. The IIP_2_ performance might result from the following factors: (1) The single-ended LNA used in this work does not suppress the even-order harmonic distortion; (2) The input of down-mixer is not exactly symmetrical, as shown in Figure 8, which causes the even-order harmonic rejection not as good as a fully differential topology. But the IIP_2_ performance of this work still meets the intermodulation requirement in BLE standard [4]. For the more severe cases not defined in [4], such as that with a large modulated out-of-band interference, a SAW filter might be needed for this work.

**Figure 15:**
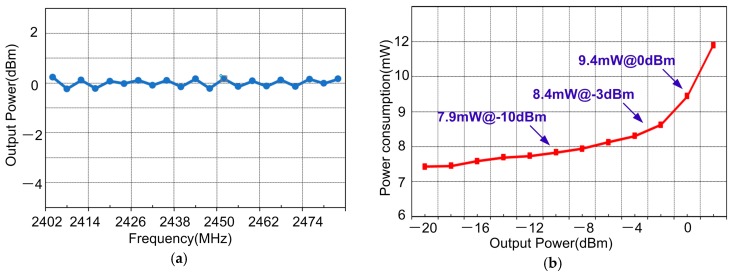
(**a**) Measured Pout variation VS ISM band frequency; (**b**) Measured power consumption vs. output power.

**Figure 16:**
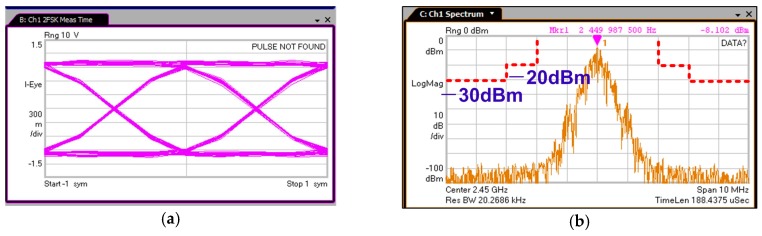

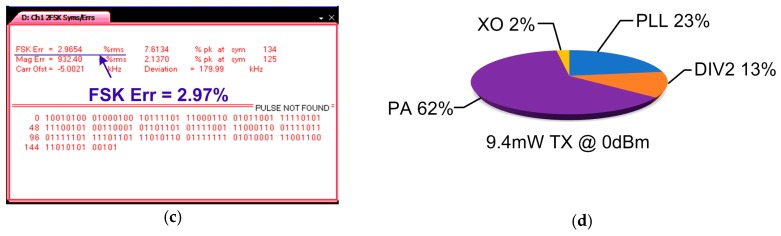
Measured TX performance. (**a**) Measured eye diagram of BLE (BT = 0.5); (**b**) Measured spectrum of BLE; (**c**) Measured FSK Error; (**d**) Power consumption summary at 0 dBm output power.

Figure 15a shows that the TX output power variations are less than 1 dB within the 2.4 GHz ISM band. The TX current consumption for various output powers is plotted in Figure 15b. It consumes 9.4, 8.4 and 7.9 mW at output powers of 0, −3 and −10 dBm, respectively. Figure 16a shows the measured eye diagram and spectrum of BLE modulation (BT = 0.5), and Figure 16b gives the in-band spurious emission performance, which is far less than the requirement in the dashed lines. The measured FSK error of BLE is 2.97% as shown in Figure 16c. All the measured modulations meet the accuracy specifications with decent margins. The power loss breakdown of TX with 0 dBm output is shown in Figure 16d. Most of the power loss is from PA, which is 5.8 mW. The last stage of the PA contributes more than 80% of this power consumption. On the other hand, the power loss on the PLL, XO and divider-by-2 circuit are similar to those in the receiver. The PA efficiency is not as high as the state-of-art works due to the following two reasons: (1) The cascode stage of PA (NM_3_) employs thick gate oxide to ensure the reliability of PA. However, the on-resistance and parasitic capacitance of thick gate oxide FET are larger than the thin gate oxide FET. Therefore, more power is consumed, comparing to the scheme using thin gate oxide FET; (2) This work employed an 110 nm CMOS process. With much larger parasitic capacitance, the switching loss is larger for this design.

**Figure 17:**
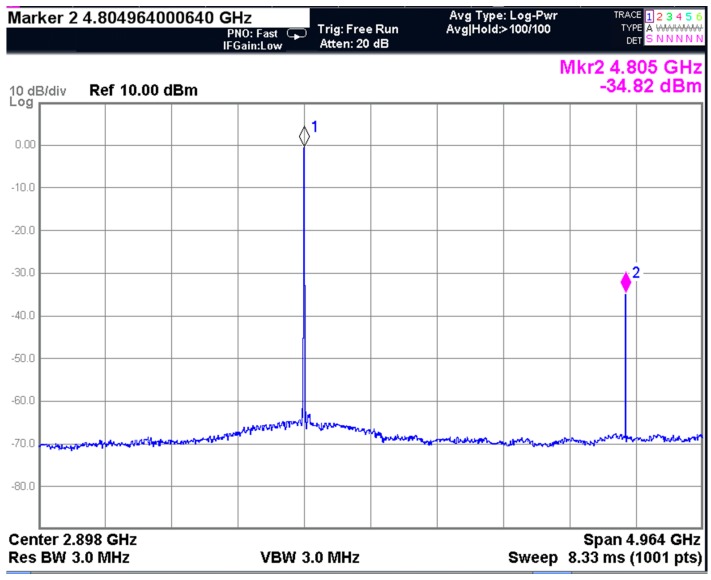
The measured output spectrum with 0 dBm required signal and −34.82 dBm 2nd harmonic spurious emission.

The measured output spectrum is presented in Figure 17, with 0 dBm required signal and −34.82 dBm 2nd harmonic emission, which meets the specification of the European standard [36] Considering the out-of-band spurious emission requirements are different in the intended countries of sales [4], more severe requirements in other standards might be employed for some specific applications. In this scenario, this design should further reject the 2nd harmonic by using a SAW filter, or reducing the output power.

**Figure 18:**
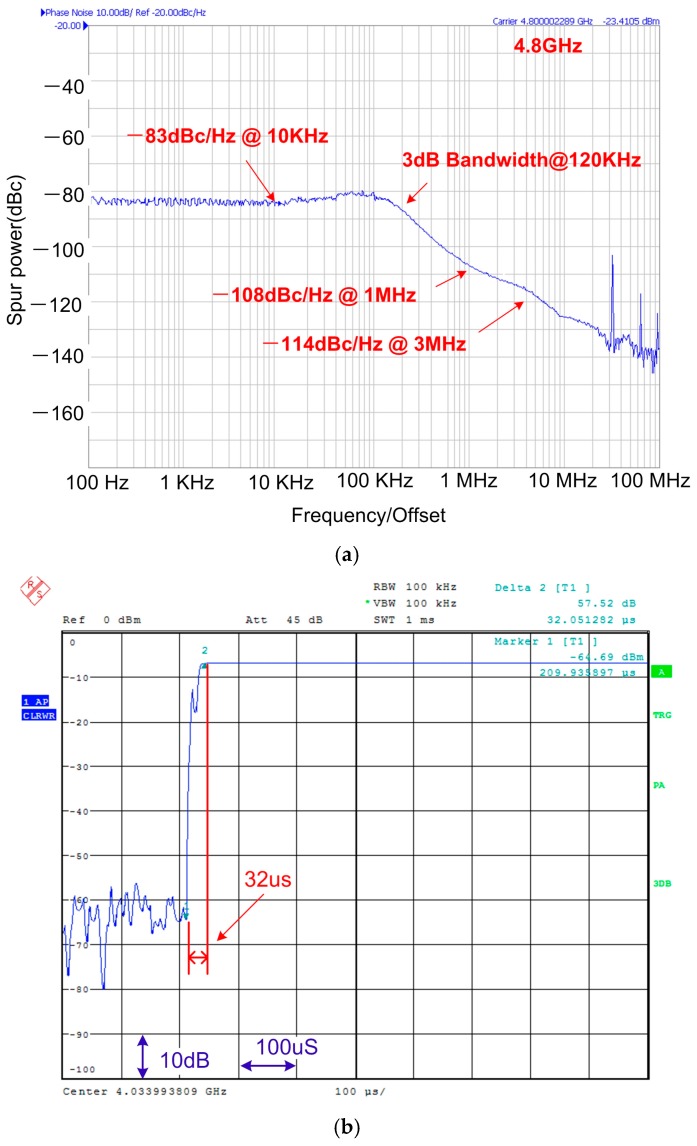
(**a**) Measured PLL phase noise at 4.8 GHz; (**b**) Measured PLL lock time.

The PLL phase noise is measured at the VCO output and the result is ploted in Figure 18a. At 4.8 GHz, the phase noises at 10 kHz, 1 MHz and 3 MHz offsets are −83 dBc/Hz, −108 dBc/Hz and −114 dBc/Hz, respectively. When applied to the LO port, the phase noise will be reduced by 6 dB due to the VCO frequency divided by 2. Therefore, the phase noise in this work is far lower than the BLE system requirements. As shown in Figure 18b, the measured PLL lock time is faster than 33 μs.

The performance of the proposed transceiver is summarized and compared with some state-of-the-art designs in Table 2. Due to the proposed front-end, only two inductors were employed in this work, and a minimum front-end silicon area is achieved. Furthermore, the TRX-switch and matching network are integrated in this work. By optimizing the impedance and matching network, and choosing a single-ended architecture for both LNA and PA, comparable S_11_, NF, output power and linearity are achieved with this proposed design. These verify the effectiveness and applicability of the proposed BLE transceiver for WSNs applications.

## 5. Conclusions

A 2.4 GHz ISM band, low cost BLE transceiver for WSNs application with RX matching network reusing PA load inductor in front-end is fabricated in a 0.11 μm CMOS technology. RX achieves a sensitivity of −93 dBm for BLE and consumes 9.7 mW. For TX, it achieves a 2.97% BLE FSK error and consumes 9.4 mW at 0 dBm output power. The front-end circuit occupies approximately 0.24 mm^2^. Measurement results verify the effectiveness and applicability of the proposed BLE transceiver for WSN applications.

## Figures and Tables

**Table 1:** IIP_3_ and IIP_2_ measurement conditions and results.

Test Mode	*f*_1_ (MHz)	*f*_2_ (MHz)	LO (MHz)	P_in_ (dBm)	Gain (dB)	Measured	Results
IIP_3_	2048.2	2051.1	2045	−50	74	P_O3_ = −41.8 dBm	IIP_3_ = −17.1 dBm
IIP_2_	2048.5	2048.2	2045	−50	74	P_O2_ = −35.8 dBm	IIP_2_ = 9.8 dBm

**Table 2:** Comparison to the state-of-art works.

	[37] nRF8001	[10] JSSC2008	[24] ISSCC2015	[25] ISSCC2015	[5] ^1^ Sensors2015	[12] VLSI2016	This Work
Compliant standards	BLE	Bluetooth	BLE	BLE	BLE/Zigbee	BLE	BLE
Data/chip rate & modulation	1 Mbps GFSK BT = 0.5	1 Mbps GFSK BT = 0.32	1 Mbps GFSK BT = 0.5	1 Mbps GFSK BT = 0.5	N.A.	1 Mbps GFSK BT = 0.5	1 Mbps GFSK BT = 0.5
Technology	N.A.	130 nm	40 nm	40 nm	180 nm	28 nm	110 nm
Number of inductors	N.A.	3	3	6	4	5	2
On chip TRX-switch	no	no	no	yes	no	yes	yes
On chip matching network	no	no	no	yes	no	yes	yes
PLL lock time (us)	130	N.A.	15	N.A.	N.A.	N.A.	32
RX sensitivity (dBm)	−87	−92	−94	−94.5	N.A.	−95	−93
RX IIP_3_ (dBm)	N.A.	N.A.	N.A.	N.A.	−19	−19	−17.1
TX max. Pout (dBm)	+4	+3	−2	0	N.A.	+3	+2
FSK error	N.A.	N.A.	4.8%	N.A.	N.A.	N.A.	2.97%
RX Power (mW)	27	36	3.3	6.3	6.74	2.75	9.7
TX Power (mW)	21 @0 dBm	33 @2 dBm	4.2 @−2 dBm	7.7 @0 dBm	N.A.	3.6 @0 dBm	9.4 @0 dBm
Front-end area (mm^2^)	N.A.	1.3 ^2^	0.5 ^2^	0.6 ^2^	2.08	1.5 ^2^	0.24

^1^ Only with RX front-end; ^2^ Estimated from respective chip microphotographs.
